# Mechanism underlying the negative effect of prostate volume on the outcome of extensive transperineal ultrasound‐guided template prostate biopsy

**DOI:** 10.1002/cam4.1300

**Published:** 2018-01-17

**Authors:** Takayoshi Demura, Takenori Takada, Naohiko Shimoda, Takaya Hioka, Yoshihumi Iwaguchi, Shin Ichihara, Hiroko Gotoda

**Affiliations:** ^1^ Department of Urology Sapporo Kosei General Hospital Sapporo Japan; ^2^ Terrestrial Ecology Graduate School of Environmental Earth Science Hokkaido University Sapporo Japan; ^3^ Department of Clinical Pathology Sapporo Kosei General Hospital Sapporo Japan

**Keywords:** Benign prostatic hyperplasia, generalized linear models, prostate volume, prostatectomy, prostatic neoplasm

## Abstract

Previous studies have indicated a possible relationship between increased prostate volume (PV) and decreased biopsy yield, although the mechanism involved is unclear. We evaluated 1650 patients who underwent template biopsy. The distribution of 993 cancer lesions in 302 prostatectomy specimens was compared with the biopsy data to determine whether each lesion was detected. A receiver operating characteristic (ROC) model was used to determine the diagnostic accuracy of prostate‐specific antigen (PSA) and related markers. A medical record number (MRN) was used as a negative control. The cancer positive rate did not change as PSA increased in patients with PV ≥50 mL (*P *= 0.466), although it increased as PSA increased in patients with PV<50 mL (*P *= 0.001). The detection rate of cancer lesions decreased as the diameter of the lesions decreased (*P *= 0.018), but remained unchanged with respect to PV. The diameters of the maximum lesions in patients with PV ≥ 50 mL were significantly smaller than those in patients with PV<50 mL (*P *= 0.003). In patients with PV ≥ 50 mL, the areas under the ROC curves for PSA‐related markers did not differ significantly from that for MRN, although they were significantly greater than that for MRN in patients with PV<50 mL (*P *< 0.001). These results suggest that an increase in PV is associated with a decrease in size and detectability of cancer lesions resulting in a decrease in biopsy yield. Loss of diagnostic accuracy of markers in patients with PV ≥ 50 mL indicates a decrease in serum levels of PSA produced by prostate cancer, which suggests growth inhibition of the cancer.

## Introduction

Previous studies have shown that prostate volume has a negative effect on the outcome of prostate biopsy 1, 2, 3. Several investigators believed that sampling error might occur in patients with larger prostate glands and that extended sampling might decrease sampling error and improve detection rate of the cancer. Although a saturation biopsy was proposed to decrease sampling error in patients with large prostates 4, 5, some reports have indicated that a saturation biopsy did not increase the cancer detection rate 6, 7. The contention that sampling error is responsible for the lower cancer detection rate in patients with larger glands remains controversial. Chen et al. 8 reported that if the lower rate of cancer detection in patients with larger prostates is due to sampling of a smaller relative volume, it would be expected that larger volume cancers would be preferentially detected in patients with larger glands. In fact, a higher proportion of smaller volume cancers were preferentially detected in patients with larger glands. They hypothesized that the lower cancer detection rate in patients with larger glands is due to ascertainment bias of prostate‐specific antigen (PSA). Patients with larger prostate glands might have higher PSA levels and more frequently undergo early prostate biopsy. The cancer detection rate of these patients is lower and the tumor size is smaller because the cancer was detected early. Recently, the association between prostate volume and high‐grade, high‐stage cancer has been reported 9, 10, 11. Freedland et al. 9 reported that prostate weight was significantly and inversely associated with the outcomes of high‐grade disease, positive surgical margins, extracapsular extension, and biochemical progression. They argued against the hypothesis of ascertainment bias of PSA since if the cancer were discovered early, patient age at the time of discovery should have been younger, but this was not the case. Hence, the contention that the lower cancer detection rate in patients with larger glands is due to ascertainment bias of PSA remains controversial. We have performed prostate biopsies using a transperineal ultrasound‐guided template method, which is a type of saturation biopsy 12, 13. In this study, we show that the lower cancer detection rate in patients with larger glands is not due to sampling error or ascertainment bias of PSA, but due to growth inhibition of the prostate cancer in larger glands.

## Materials and Methods

### Template biopsy method

We evaluated 1650 men who underwent transperineal template biopsy at our hospital between September 2000 and June 2017. Locally advanced cancers (T3 and T4) and metastatic cancers were excluded from this study since these patients usually underwent transrectal or transperineal sextant biopsy in our hospital. Furthermore, in the case of advanced cancers, it is difficult to determine whether the cancer in a large prostate gland grew or the cancer in a small gland grew to a large advanced cancer. For cases with multiple template biopsies only the latest data was used. Serum levels of total and free PSA were measured using the Abbott IMx assay. Prostate volume was estimated using transrectal ultrasonography with the following equation; π/6 × length × width × height, with length being measured in the longitudinal view and width and height being measured in the transaxial view. PSA density (PSAD) was calculated by dividing the serum PSA concentration by the prostate volume (expressed in mL). All patients underwent systematic ultrasound guided biopsy using the transperineal template technique as previously described 12. Following digital rectal examination (DRE), a transrectal ultrasound probe covered with a standard condom containing scanning gel was inserted into the patient's rectum. Subsequently, the probe was mounted in the stepping unit. The prostate was scanned transversely from the base to the apex using the stepping unit. A step size was 5 mm. The matrix pattern was superimposed onto the transverse image 1 cm proximal from the apex. The 18‐gauge Tru‐Cut biopsy needle was inserted through the corresponding guide channel of the template, and a mean of 21 biopsy cores ranging from 9 to 43 according to the prostate volume were taken in each biopsy set. Biopsy specimens were individually labeled and fixed in formalin, embedded in paraffin, and then stained with hematoxylin and eosin.

We evaluated prostatectomy specimens from the 302 patients who underwent radical prostatectomy in our hospital without previous hormonal therapy. Whole‐mount sectioning of each prostatectomy specimen and detailed morphometric mapping were performed at 4–5 mm intervals and at sagittal sections for proximal and distal ends of the specimen. We compared the distribution of cancer lesions in the prostatectomy specimen with the template biopsy data to determine whether each lesion was detected by the biopsy. The diameter of each cancer lesion was determined as a mean of the longitudinal and lateral length of the cancer lesion.

### Statistical analysis

The Mann–Whitney *U*‐test was used to determine the significance of differences in two groups, while the Kruskal–Wallis test was used to determine the significance of differences in more than two groups, and the Steel‐Dwass test was used as a post hoc test. Fisher's exact test (extended) was used for categorical comparison of the data. Pearson's Coefficient of Correlation was used to detect if there was any association between the two variables. Generalized linear models (GLMs) were used for the multivariate analysis using R statistics version 3.3.2. 14. The odds ratio was expressed as the unit odds ratio. A receiver operating characteristic (ROC) model was used to determine the diagnostic accuracy of tumor markers on the outcome of the biopsy. The significance of the difference in the area under the ROC curve (AUC) was determined using a bootstrap method. All *P* values were two‐sided and *P *<* *0.05 was considered statistically significant.

## Results

### Prostate volume and biopsy yield

**Figure 1:**
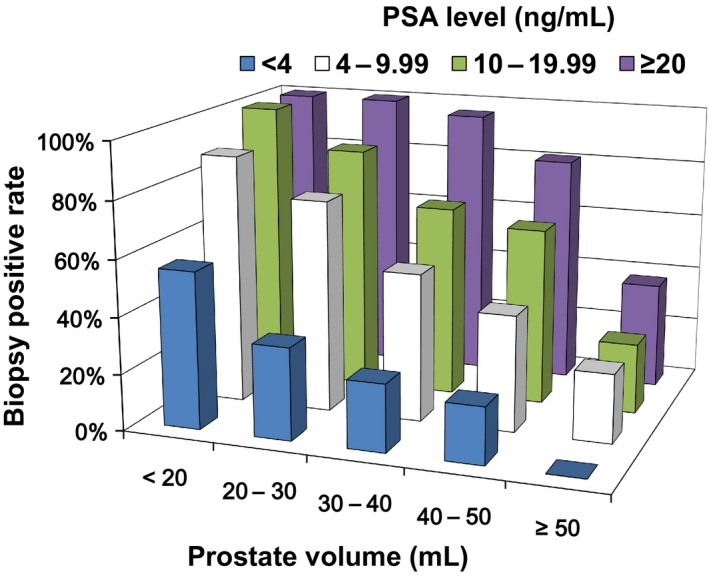
Cancer positive rates of template biopsy in groups stratified according to levels of prostate‐specific antigen (PSA) and prostate volume.

Among the 1650 men considered in this study, 969 (59%) were diagnosed as having prostate cancer. The median PSA level of the 1650 men was 7.18 ng/mL (interquartile range (IQR); 5.17–10.84). Table 1 shows descriptive statistics comparing patients with cancer and those with no cancer on template biopsy. Patients with prostate cancer were significantly older (*P *< 0.001), had higher PSA levels (*P *< 0.001), PSAD levels (*P *< 0.001), smaller prostate glands (*P *< 0.001), and more frequently had positive DRE findings (*P *< 0.001) than those with no cancer. Cancer positive rates of template biopsy in the groups stratified according to PSA level and prostate volume are shown in Figure 1 and Table 2. When subjects were stratified according to PSA level (<4, 4–9.99, 10–19.99 and ≥20 ng/mL), the cancer positive rate decreased significantly as the prostate volume increased in groups with PSA levels ≥4 ng/mL (*P *<* *0.001 for all). However, the cancer positive rate did not change as the prostate volume increased in the group with PSA levels <4 ng/mL (*P *= 0.365). When subjects were stratified according to prostate volume (<20, 20–30, 30–40, 40–50 and ≥50 mL), the cancer positive rate increased significantly as PSA increased in groups with prostate volume <50 mL (*P *=* *0.001 or <0.001). However, the cancer positive rate did not change significantly as PSA increased in the group with prostate volume ≥50 mL (*P *= 0.466).

**Table 1:** Clinical characteristics of patients with and without prostate cancer on template biopsy

Characteristics	Patients with cancer (*n* = 969)	Patients with no cancer (*n* = 681)	*P*‐value
	Median (IQR)	Median (IQR)	
Age	70 (64–76)	67 (62–72)	<0.001
PSA (ng/mL)	7.71 (5.46–12.11)	6.67 (4.85–9.42)	<0.000
PSAD	0.268 (0.177–0.458)	0.160 (0.118–0.225)	<0.001
Prostate volume (mL)	28.3 (22.5–37.3)	40.7 (32.1–55.6)	<0.001
DRE finding positive	337	130	<0.001
Negative	632	551	

IQR, interquartile range; PSA, prostate‐specific antigen; PSAD, PSA density; DRE, digital rectal examination.

**Table 2:** Cancer positive rates of template biopsy in groups stratified according to levels of prostate‐specific antigen (PSA) and prostate volume

Prostate volume (mL)	PSA (ng/mL)								*P*‐value
	<4		4–9.99		10–19.99		≥20		
	No. of patients								
	Cancer (%)	Total	Cancer (%)	Total	Cancer (%)	Total	Cancer (%)	Total	
<20	5 (56%)	9	100 (88%)	113	40 (100%)	40	13 (100%)	13	0.001
20–30	14 (33%)	43	232 (75%)	309	83 (86%)	96	42 (100%)	42	<0.001
30–40	5 (24%)	21	158 (52%)	302	57 (68%)	84	23 (96%)	24	<0.001
40–50	3 (20%)	15	70 (41%)	171	32 (63%)	51	17 (81%)	21	<0.001
≥50	0 (0%)	2	44 (24%)	181	22 (25%)	89	9 (38%)	24	0.466
*P*‐value	0.365		<0.001		<0.001		<0.001		

PSA, prostate‐specific antigen.

### Prostate volume and cancer lesions in prostatectomy specimens

We found 993 cancer lesions in 302 prostatectomy specimens. Of the 993 lesions, 505 were detected with template biopsy. A multivariate logistic regression model was generated to predict the detection of cancer lesions as a function of patient age, PSA, prostate volume, Gleason score of the cancer lesion, and diameter of the maximum cancer lesion. We analyzed with only the maximum cancer lesion in each prostatectomy specimen to solve the problem on duplication of explanatory variables. On multivariate logistic regression analysis, the Gleason score of the maximum cancer lesion and the diameter of the maximum lesion were identified as a predictor of cancer detectability by template biopsy (*P *= 0.016 and 0.018, respectively), however, the prostate volume as well as PSA and patient age had no influence (*P *= 0.585, 0.855 and 0.303, respectively) (Table 3, Model‐A).

**Table 3:** Multivariate logistic regression model to predict detection of the maximum cancer lesions as a function of patient age, prostate‐specific antigen, prostate volume, Gleason score of the maximum cancer lesion, and diameter of the maximum cancer lesion

	Odds ratio (95% CI)	*P*‐value
Model‐A		
Age	1.050 (0.960–1.140)	0.303
PSA	0.989 (0.878–1.110)	0.855
Prostate volume (a continuous variable)	0.990 (0.956–1.030)	0.585
Gleason score of the cancer lesion	2.71 (1.20–6.13)	0.016
Diameter of the maximum cancer lesion	1.54 (1.45–1.63)	0.018
Model‐B		
Age	1.050 (0.963–1.140)	0.274
PSA	0.983 (0.876–1.100)	0.767
Prostate volume (a binary variable of <50 mL and ≥50 mL)	1.45 (0.27–7.84)	0.663
Gleason score of the cancer lesion	2.81 (1.24–6.38)	0.013
Diameter of the maximum cancer lesion	1.16 (1.03–1.30)	0.012

Model‐A was generated by incorporating prostatic volume as a continuous variable. Model‐B was generated by incorporating prostatic volume as a binary variable of <50 mL and ≥50 mL. CI, confidence interval; PSA, prostate‐specific antigen.

**Figure 2:**
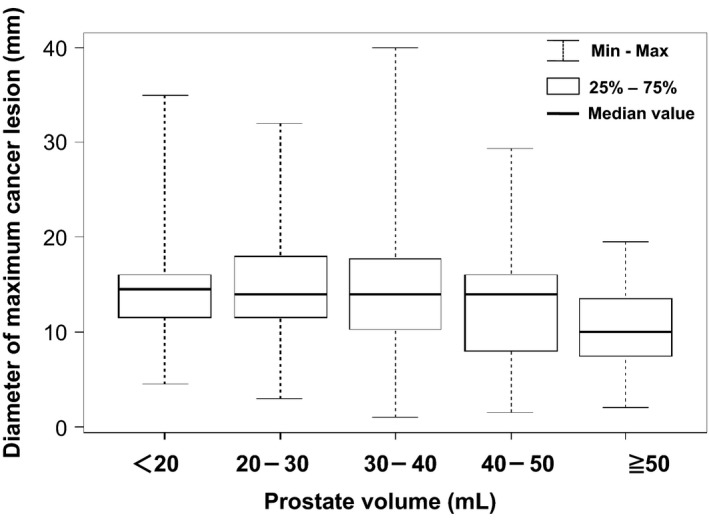
Box and whisker plots show the diameters of maximum cancer lesions in five groups separated on the basis of prostate volume.

Then we evaluated the association between prostate volume and diameter of the maximum cancer lesion in the prostatectomy specimen. We divided 302 prostatectomy specimens into five groups based on prostate volume (<20, 20–30, 30–40, 40–50 and ≥50 mL). The median diameter of the maximum cancer lesion in each group with prostate volume <50 mL ranged from 14.0 to 14.5 mm, while that for the group with prostate volume ≥50 mL was 10.0 mm (Fig. 2 and Table 4). The hypothesis suggesting that the diameter of the maximum cancer lesions in all groups was the same was not supported (*P *= 0.018). The diameter of the maximum lesion in the group with prostate volume ≥50 mL was significantly less than that in the group with prostate volume <20 mL (*P *= 0.033) and in the group with prostate volume ranging from 20 to 30 mL (*P *= 0.015) with a post hoc test. The diameter of the maximum lesion in patients with prostate volume ≥50 mL was significantly less than that in patients with prostate volume <50 mL (*P *= 0.003). These results show that prostate volume is clearly divided in <50 and ≥50 mL. To predict the detection of cancer lesions, we generated another multivariate logistic regression model (Model‐B), which incorporated prostate volume as a binary variable of <50 and ≥50 mL. On multivariate logistic regression analysis, the Gleason score of the maximum cancer lesion and the diameter of the maximum lesion were identified as a predictor of cancer detectability (*P* = 0.013 and 0.012, respectively) although the prostate volume as well as PSA and patient age had no influence (Table 3, Model‐B). The number of cancer lesions in the prostatectomy specimen in five groups separated on the basis of prostate volume (<20, 20–30, 30–40, 40–50, and ≥50 mL) was compared and found not to differ significantly (*P *= 0.929) (Table 4).

**Table 4:** Diameter of the maximum cancer lesion and number of cancer lesions in the prostatectomy specimen in five groups separated on the basis of prostate volume

Prostate volume (mL)	No. of patients	Diameter of the maximum cancer lesion (mm)	No. of cancer lesions
		Median (IQR)	Median (IQR)
<20	46	14.5 (11.5–16.0)	3 (2–4)
20–30	115	14.0 (11.6–18.0)	3 (2–4.75)
30–40	87	14.0 (10.3–17.8)	3 (2–4)
40–50	33	14.0 (8.0–16.0)	3 (1–4)
≥50	21	10.0 (7.5–13.5)	3 (2–4)

IQR, interquartile range.

### Prostate volume and patient age

**Figure 3:**
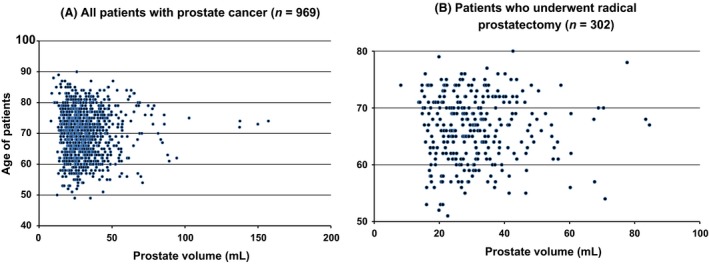
Scatter plot shows the relationship between prostate volume and age of patients with prostate cancer (*n* = 969) (A) and patients who underwent radical prostatectomy (*n* = 302) (B).

The relationship between prostate volume and age of patients with prostate cancer (*n* = 969) is shown in the scatter plot (Fig. 3A). There was no correlation between age and prostate volume (coefficient 0.036, 95% confidence interval (CI) −0.027–0.098, *P *=* *0.267). The relationship between prostate volume and age of patients who underwent radical prostatectomy (*n* = 302) is also shown in the scatter plot (Fig. 3B). There was no correlation between age and prostate volume (coefficient 0.008, 95% CI −0.105–0.121, *P *=* *0.891) in those patients.

### Prostate volume and diagnostic accuracy of PSA and related markers

**Figure 4:**
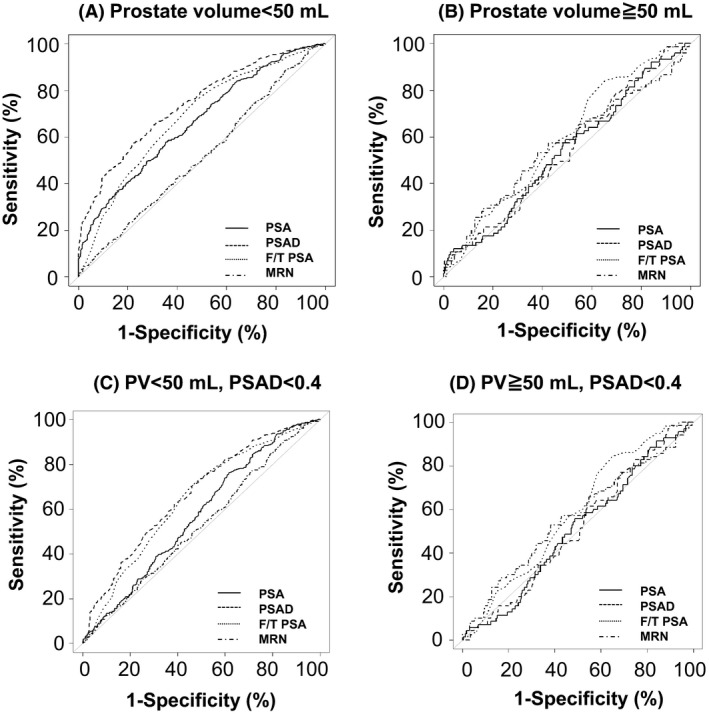
Receiver operating characteristic curves for prostate‐specific antigen (PSA), PSA density (PSAD), free/total PSA ratio (F/T PSA) and medical record number of each patient in our hospital (MRN) as a negative control in patients with prostate volume less than 50 mL (A), in those with prostate volume of at least 50 mL (B), in those with prostate volume less than 50 mL and PSAD less than 0.4 (C), and in those with prostate volume of at least 50 mL and PSAD less than 0.4 (D) are shown.

We compared the diagnostic accuracy of PSA and related markers for patients with prostate volume <50 mL to that of patients with prostate volume ≥50 mL. The ROC curves for PSA, PSAD and the free/total PSA ratio (F/T PSA) are shown in Figure 4, and the area under the ROC curve (AUC) for each marker is shown in Table 5. The ROC curve for medical record number of each patient in our hospital (MRN) was used as a negative control. In patients with prostate volume <50 mL, the AUCs for PSA, PSAD, and F/T PSA were significantly greater than the AUC for MRN (*P *< 0.001 for all) (Fig. 4A). In those patients, the AUC for PSAD was significantly greater than the AUCs for PSA and F/T PSA (*P *< 0.001 for both). However, in patients with prostate volume ≥50 mL, the AUCs for PSA, PSAD, and F/T PSA did not differ significantly from the AUC for MRN (*P* = 0.638, 0.642 and 0.387, respectively) (Fig. 4B). In 296 patients with prostate volume ≥50 mL, 289 (98%) have PSAD <0.4. In patients with prostate volume ≥50 mL and PSAD <0.4, the AUCs for PSA, PSAD, and F/T PSA did not differ significantly from the AUC for MRN (*P *= 0.331, 0.149 and 0.497, respectively) (Fig. 4D). However, in patients with prostate volume <50 mL and PSAD <0.4, the AUCs for PSAD and F/T PSA were significantly greater than the AUC for MRN (*P *< 0.001 for both), although the AUC for PSA did not differ significantly from that for MRN (*P *=* *0.096) (Fig. 4C).

**Table 5:** Areas under the receiver operating characteristic curves are shown for all patients and patients with prostate‐specific antigen density levels less than 0.4

Markers	PV < 50 mL	PV ≥ 50 mL
	AUC (95% CI)	AUC (95% CI)
All patients		
PSA	0.663 (0.634–0.693)	0.534 (0.459–0.609)
PSAD	0.741 (0.715–0.768)	0.540 (0.466–0.614)
F/T PSA	0.682 (0.651–0.713)	0.591 (0.515–0.668)
MRN	0.517 (0.484–0.550)	0.561 (0.482–0.640)
Patients with PSAD < 0.4		
PSA	0.571 (0.535–0.607)	0.506 (0.431–0.581)
PSAD	0.666 (0.632–0.699)	0.512 (0.438–0.585)
F/T PSA	0.643 (0.608–0.678)	0.582 (0.504–0.659)
MRN	0.526 (0.491–0.562)	0.562 (0.482–0.642)

Each group is divided into two groups according to prostate volume (<50 mL and ≥50 mL). PV, prostate volume; AUC, area under the receiver operating characteristic curve; CI, confidence interval; PSA, prostate‐specific antigen; PSAD, prostate‐specific antigen density; F/T PSA, free/total PSA ratio; MRN, medical record number of each patient in our hospital.

## Discussion

In this study, we showed that the biopsy positive rate decreased as the prostate volume increased in patients with PSA levels greater than 4 ng/mL, and that the detection rate of cancer lesions in the prostatectomy specimens did not change according to prostate volume, although it decreased with decreasing diameter of the lesion. On multivariate logistic regression analysis, diameter of the maximum lesion was identified as a predictor of cancer detectability, although the prostate volume was not a significant factor. To evaluate the effect of an interaction between each explanatory variable, we analyzed the interaction using GLMs by incorporation it into the model equation as the product of the variables. These analyses showed no interaction between each variable. In the model selection (using R statistics stepwise command with selection criteria of Bayesian Information Criterion, BIC), only the diameter of maximum cancer lesion and Gleason score of the lesion remained (data not shown), which is considered that there is no interaction between each variable. These results suggest that although template biopsy can prevent sampling error in patients with large prostate glands, it is unable to prevent sampling error in those cases with small cancer lesions. We showed that the diameter of the maximum cancer lesion in patients with prostate volume ≥50 mL was significantly less than that in patients with prostate volume <50 mL. These results suggest that an increase in prostate volume causes a decrease in the size of the maximum cancer lesion and a decrease in the detectability of the lesion, thereby resulting in a decreased yield of template biopsy in patients with large glands (≥50 mL). Chen et al. 8 reported that small‐volume cancers (0.5 cc or less) were twice as frequent in large prostate glands (≥50 g), which is consistent with our results. Two hypotheses are considered to explain this phenomenon, namely ascertainment bias because of the performance characteristics of PSA, and true tumor biology. If the cancer lesions were small as they were discovered early in patients with larger glands, the patients should be younger. However, our analysis showed no correlation between age and prostate volume in all patients with prostate cancer or in patients who underwent radical prostatectomy, which is consistent with the results described by Freedland et al. 9. The size of the cancer lesion in the prostatectomy specimen is the average growth rate multiplied by the growth time from the occurrence of the cancer to prostatectomy. Therefore, a small cancer lesion indicates slow growth rate or short growth time. If there is no difference in patient age at the time of prostatectomy, it is suggested that there is no difference in the growth time, or the growth time is short because the cancer has occurred recently. The former indicates slow growth rate of the cancer, the latter indicates that cancer occurrence is inhibited until a patient becomes older. In either case, it is suggested that the occurrence or growth of prostate cancer is inhibited in large glands. If cancer occurrence was inhibited, the number of cancer lesions in the prostatectomy specimen would decrease, however, we showed that the number of cancer lesions in the prostatectomy specimen did not change as the prostate volume increased. Therefore, it is probable that the growth rate of the cancers is slow in patients with larger glands (≥50 mL). If the growth of prostate cancer is suppressed in those patients, serum levels of PSA produced by the cancer may decrease and the use of PSA‐related markers may be invalid for those patients. We showed that in patients with prostate volume ≥50 mL, the AUCs for PSA, PSAD, and F/T PSA did not differ significantly from the AUC for MRN (medical record number of each patient in our hospital), although those were significantly greater than the AUC for MRN in patients with prostate volume <50 mL. These results indicate that the proportion of serum PSA produced by prostate cancer is low in patients with prostate volume ≥50 mL. There is also the possibility that the diagnostic accuracy of PSA‐related markers is lowered because the cancer is found early. The patients with prostate volume ≥50 mL have low levels of PSAD. Ninety‐eight percent of those patients have PSAD levels <0.4. We investigated whether the diagnostic accuracy of PSA and related markers also declined in patients with PSAD <0.4 and prostate volume <50 mL. In those patients, the AUCs for PSAD and F/T PSA were significantly greater than that for MRN, suggesting it is unlikely that the diagnostic accuracy of tumor markers is lowered because of detecting cancer at the early stage. Thus, we hypothesized the growth of prostate cancer is inhibited in large glands (≥50 mL) and benign prostatic hyperplasia (BPH) may inhibit the growth.

The following process is hypothesized as a mechanism to account for the occurrence of BPH, where inflammation occurs, cytokines and chemokines are produced, and expression of growth factors causes prostate proliferation and angiogenesis 15. If BPH suppresses the proliferation of prostate cancer, the growth factor promoting the growth of BPH should simultaneously inhibit cancer growth. Yang et al. 16 reported that when placental growth factor (PlGF) was expressed in a tumor not expressing vascular endothelial growth factor (VEGF), tumor angiogenesis was promoted and the tumor grew, but when PlGF was expressed in the tumor expressing VEGF, tumor angiogenesis was inhibited and tumor growth was inhibited. In the absence of VEGF, PlGF forms a homodimer and binds to the VEGF receptor (VEGFR) ‐1 to promote angiogenesis, but in the presence of VEGF, PlGF forms a heterodimer with VEGF, thereby inhibiting the binding of VEGF and VEGFR‐2 and inhibiting angiogenesis. In the prostate, VEGF is highly expressed in the cancer 17 and PlGF is in BPH 18. Furusato et al. 19 measured angiogenesis of prostate cancer with blood capillary density ratios and investigated the relationship with tumor volume in autopsy cases. They demonstrated that when the tumor volume exceeded 83 mm^3^ (assuming a spherical shape, the tumor diameter was 5.4 mm), angiogenesis increased and the tumor increased. From these results, the following mechanism was postulated. Initially, when cancer occurs in the prostate gland, the cancer is small and considered to be in a latent state. As the tumor grows to a diameter of about 5 mm, VEGF is expressed and cancerous angiogenesis occurs. At that time, if a large amount of PlGF is released from the BPH tissue around the cancer, it forms a heterodimer with VEGF derived from the cancer, inhibits the binding of VEGF with VEGFR‐2, and subsequently inhibits angiogenesis and cancer proliferation. If angiogenesis in the prostate cancer is inhibited, the passage of PSA secreted by the cancer into the blood should be suppressed, and the use of PSA and related markers would be invalid for the diagnosis of prostate cancer.

However, this study has several limitations. It is a case–control study and may include selection bias. The prostatectomy specimens in this study had at least a cancer lesion detected using template biopsy. The cancer not detected using the biopsy may have influenced the result. Furthermore, we speculated that growth time from the occurrence of the cancer to prostatectomy was similar despite the prostate volume increased, however, in clinical research, we cannot know when the cancer occurred actually. Basic research on the relationship between prostate cancer and BPH is required in an effort to understand the relationship between prostate volume and size of cancer lesions.

## Conflict of Interest

The authors have no disclosures.
